# Supplementary material for: Patterns of Intron Gain and Loss in Fungi
Source: PLoS Biol. 2004 Nov 30;2(12):e422. doi: 10.1371/journal.pbio.0020422 (PMC532390; doi:10.1371/journal.pbio.0020422)
Supplement: Table S1 — Also available at http://genes.mit.edu/NielsenEtAl/. (4.3 MB ZIP). [file pbio.0020422.st001.zip › NielsenEtAl/html/21.html]

AN1676.1.NCU04260.1.MG00953.1.FG10392.1


```
 CLUSTAL W (1.82) Multiple Sequence Alignments - Introns Inserted


Sequence 1: MG00953.1	342 aa
Sequence 2: FG10392.1	266 aa
Sequence 3: NCU04260.1	405 aa
Sequence 4: AN1676.1	279 aa
Alignment Length: 451 aa
Number Identitical Residues: 55 aa
Alignment Score (without introns) 3640


MG00953.1 	---------MASIPDPDLEFSDDEQQDRSFRPFKVVYNFAPVPIPDSFLSTANPPPDAQP
NCU04260.1	MSSVLAELQKLVTPTPPHQPQPKNKAHHQSNPIKTTYESLPIDLPPDFLAT---EPGITP
FG10392.1 	------------------------------MPQKIEYNSNDIPIPDSFLTS----PSQTP
AN1676.1  	----------------------------------MAFG------KDQFLPS---QPPANA
          	                                    :         .**.:    *   .

MG00953.1 	V-TLTPVDFS-SVLPEYQGCHAVVLENVLSPSECVQLLRMAEASVPEADRYERRVKKKPR
NCU04260.1	VPTLTEIDWATTDLPENKGLYAVILDEVLTKSECDELRKLAEGSVPLS------------
FG10392.1 	I-TSHQIDFANSPLPQYDGHTALVLDNVISPAECRELLSLAEASVPR-------------
AN1676.1  	RCRVIDFASTEPPLPEYKRYLAAVIDNALTETECKELLRLAEASTADG------------
          	      .  : . **: .   * ::::.:: :** :*  :**.*.. .            

MG00953.1 	RSSSSSSSGKQSTDDASSSPPTAPEVFRDPWRPACVSLGANLEILDRGYRRGDRIVWDEQ
NCU04260.1	----------QWVDVDGQEQDGATTKKTTPWAPALVNVGLGYEVLTPSYRNSSRIIWDQQ
FG10392.1 	-------------DDESQS----------AWKPALVSGGDGYETRAPGYRESDRIIWDQQ
AN1676.1  	-----------------------------KWERAMINVGGGRQKISTDSRNSSRIILDSP
          	                              *  * :. * . :    . *...**: *. 

MG00953.1 	ELTDRLWARCVQAPGLLEQLAFIGPDD------------PAAPFKSR-------------
NCU04260.1	EVVDRLWKRCCLVPGLMERLAVLDAGRERDVKIITGRSLPSQPAAAVGDEGDGQDGKQEE
FG10392.1 	TIVDRLWERCLQADGLRDLLAVV----------------PHEPWMK--------------
AN1676.1  	EIAEKLQARLLPFLRELEVDRLE-----------------NKPLITG-------------
          	 :.::*  *        :   .                    *                 

MG00953.1 	--------------------------YSANPPWRFSRINKRMRFLK~YRRGDFFRP1HCD
NCU04260.1	KKEVVERYANGKKKGKSKRTKTKGLRDGRTGKWEFVKVNKRMRFLR~YGEGQFFRA1HCD
FG10392.1 	-----------------------------GGKWVFSRLNDRMRFLK~YSPGQYFKP1HCD
AN1676.1  	-------------------------LAGRNRTYKFTRLNERLRFLK0YVGGEYFRP~HWD
          	                           .    : * ::*.*:***: *  *::*:. * *

MG00953.1 	GAY--EDS-SHGKQRQTLYTLHLYLNDSKVEAAMAGREPTDLVG----------------
NCU04260.1	SPYRELDPINSDRINETLFTIHLYLNDCKSEAPPAKKDETELVG----------------
FG10392.1 	GAY--FYTEGPGKEFETFYTVHLYLNDS-----AENDPASELQG----------------
AN1676.1  	AHY-----TTPDRRERSYFTIQLYLNGDGEQDLQELKCAIDAEGPEGSERGCMNLDVEGR
          	. *        .:  .: :*::****.   .         :  *...:. .. . . .. 

MG00953.1 	---GSTPFLS--KDGSRRIDVNPRCGRVLIFQHNKLRHSGDDVVYGVKYTMRTDLLYEMV
NCU04260.1	---GATALLS--GDEKRKYDVECKTGRVLIFQHRRVFHAGADVVKGVKYSVRTDIMYREV
FG10392.1 	---GATSFLD--RRGEKRVDVNPKAGSVLIFQHKGLFHEGALVNKGVKYTMRTDILYEWI
AN1676.1  	LLGGATSFLPRYEEKERHVRIFPRTGSALVFQQSELLHSGDPVFRGTKFTLRMDVMYELV
          	  .*:*.:*      .::  :  : * .*:**:  : * *  *  *.*:::* *::*. :

MG00953.1 	GPEEQEKRSK-----------------------
NCU04260.1	LDEPEEKEVGEGNEQAEKKADDDKPLKEEDDGK
FG10392.1 	ADKDEE---------------------------
AN1676.1  	ESESY----------------------------
          	  :
```
